# Health financing, digital connectivity, and universal health coverage: a country-year panel study

**DOI:** 10.3389/fpubh.2026.1928583

**Published:** 2026-10-09

**Authors:** Guoliang Lu, Xinyun Tang

**Affiliations:** 1 School of Journalism and Communication, Chongqing University, Chongqing, China; 2 Institute of Ecological Sociology, Fujian Academy of Social Sciences, Fuzhou, China; 3 School of Journalism and Communication, Shanghai International Studies University, Shanghai, China

**Keywords:** country-year panel, digital connectivity, digital public health, governance, health financing, information environment, out-of-pocket expenditure, universal health coverage

## Abstract

**Introduction:**

Universal health coverage (UHC) requires broad service coverage and financial protection. Digital connectivity may enable digital public health, but general internet and telecommunications access does not capture the adoption, integration, quality, or equitable use of digital health services. We examined how health financing, connectivity, governance, and online information environments relate to UHC outcomes.

**Methods:**

We assembled a multi-source country-year panel spanning 2000–2024. The principal two-way fixed-effects model included 3,522 observations from 166 countries in 2001 to 2023, with one-year-lagged exposures, country and year fixed effects, and country-clustered standard errors. Supplementary analyses used WHO report-reference-year and interval-change models, joint lag–lead diagnostics, an expanded health-supply composite, a 2023 Global Digital Health Monitor (GDHM) construct comparison, post-2020 tests, and false discovery rate (FDR)-controlled exploratory outcomes.

**Results:**

A 1-SD higher out-of-pocket expenditure share was associated with a 1.38-point lower UHC service coverage index (95% CI, −2.39 to −0.36), whereas government health expenditure share was not independently associated with the index. A 1-SD higher connectivity composite was associated with a 2.98-point lower index (95% CI, −4.05 to −1.91) in the principal level model. However, this association attenuated across alternative estimands and was null for three-year change (−0.17; 95% CI, −0.66 to 0.32) and annualized change between WHO reference years (−0.17; 95% CI, −0.38 to 0.04). FDR-controlled analyses showed heterogeneous domain-specific patterns: connectivity was associated with lower DTP3 and MCV1 coverage but lower neonatal mortality, whereas public financing was associated with higher MCV2 coverage. UHC interactions were null, while immunization interactions varied by government effectiveness and information environment.

**Discussion:**

Connectivity was associated with UHC levels, whereas the three-year and WHO reference-interval change estimates had confidence intervals spanning zero; associations also varied across domain-specific outcomes. Because the exposure captures general infrastructure rather than digital-health implementation, the findings suggest that connectivity alone is insufficient to improve UHC. Its public-health contribution is likely conditioned by financing, governance, organizational capacity, interoperability, workforce readiness, and equitable uptake. Causal interpretation is limited by the ecological design, modeled and interpolated UHC values, and unmeasured health-system characteristics.

## 1 Introduction

Universal health coverage combines access to needed, high-quality services with protection against financial hardship. These dimensions are related but not interchangeable: a country can expand tracer-service coverage while households remain exposed to unaffordable out-of-pocket payments, or improve financial protection without uniformly expanding service delivery (1–3). Health financing therefore matters not only through the volume of resources but also through pooling, purchasing, equity, and accountability (4, 5, 9).

Digital technologies are increasingly embedded in public-health surveillance, risk communication, clinical support, supply chains, and service delivery. Cross-country studies often operationalize this environment with internet penetration, mobile subscriptions, or related information and communication technology indicators (6–8). Such indicators are informative about national connectivity, but they do not reveal whether countries have deployed interoperable records, telemedicine, clinical decision support, mature health-information systems, trained workforces, or accessible digital services.

This distinction is central. WHO guidance treats digital interventions as health-system interventions whose benefits depend on functional services, governance, architecture, financing, implementation planning, and equity (10, 11). Connectivity can make digital health possible, but it is only the first element in a longer pathway: connectivity to adoption and integration; adoption to usable service capacity; and service capacity to equitable utilization and health outcomes. General connectivity and implemented digital-health services should therefore be treated as related but distinct constructs.

Information environments may shape a second pathway. Online disinformation, fragmentation, pluralism, trust, and political communication can affect the credibility and reach of public-health messages (12–21). The Digital Society Project (DSP), however, measures national political and media environments rather than direct exposure to health misinformation. Its indicators should therefore be treated as contextual moderators, not as individual-level measures of misinformation exposure.

Health-system organization may condition these pathways. Classic labels such as tax-funded national services, social health insurance, and private insurance obscure hybrid arrangements, segmentation, pooling, purchaser-provider relationships, and changes over time (22, 23). A globally harmonized annual typology is not available for the full study period, and time-invariant labels are absorbed by country fixed effects. In this study, financing shares represent financing structure, while workforce and hospital-bed indicators capture selected dimensions of supply capacity.

The COVID-19 pandemic further altered the opportunity structure for digitally mediated care. Telemedicine, remote triage, digital surveillance, vaccination platforms, and online risk communication expanded rapidly, while essential services were disrupted (15, 24, 25). We therefore examined whether the connectivity-UHC association differed after 2020.

We investigated how health financing, general digital connectivity, governance, and macro-level information environments were associated with UHC service coverage and financial protection in a longitudinal cross-national panel. The principal analysis assessed one-year-lagged associations with the UHC service coverage index. We then examined scale construction, within-country variation, post-2020 change, alternative lag structures and estimands, health-system supply measures, financing composition, income groups, and common-sample interactions. Immunization, preparedness, mortality, and financial-protection models were designated exploratory and evaluated with false-discovery-rate control. Figure 1 presents the conceptual framework linking general digital connectivity with service coverage and health outcomes.

**Figure 1:**
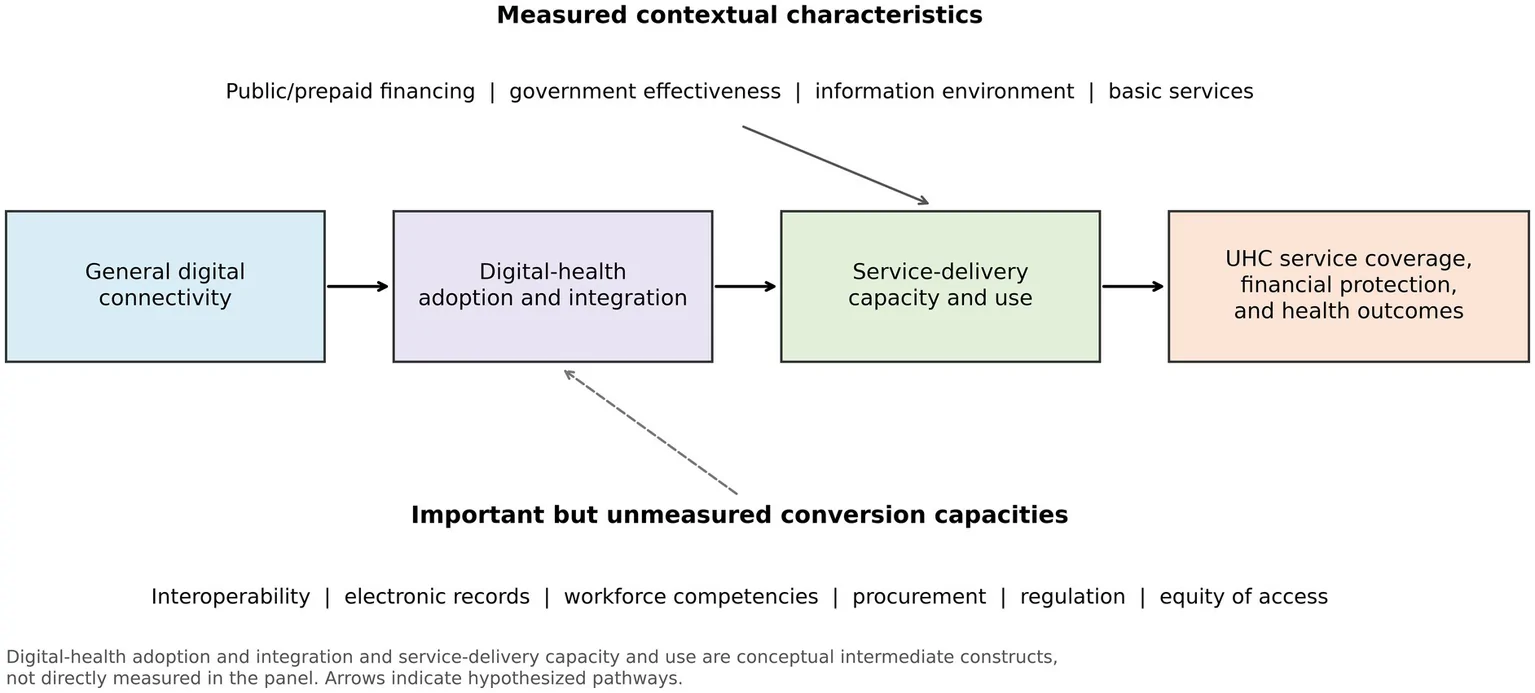
Conditional conversion framework. General connectivity is an enabling infrastructure rather than direct digital-health implementation. Financing, health-system organization, governance, information environments, workforce and implementation capacity, and equitable access may determine whether infrastructure is converted into service coverage and population-health gains. Digital-health adoption and integration and service-delivery capacity and use were not directly measured in the panel; the arrows depict hypothesized pathways.

## 2 Materials and methods

### 2.1 Study design and reporting

We conducted an ecological country-year panel study using publicly available aggregate data. The source panel covers 2000–2024, while the principal UHC model covers 2001–2023 because exposures were lagged one year and the WHO UHC series ended in 2023. The analysis was not preregistered. The UHC model was treated as the principal analysis; outcome extensions and interaction models were treated as exploratory. Reporting follows the STROBE framework for observational studies (26).

### 2.2 Data sources and linkage

Country-year records were linked by ISO3 code and year. Sources included the WHO Global Health Expenditure Database and Global Health Observatory, World Bank World Development Indicators and Worldwide Governance Indicators, telecommunications indicators used to represent general connectivity, and DSP/V-Dem measures of online political communication (27–34). The principal UHC model included countries with at least two complete observations. Secondary models used outcome-specific complete cases, including countries with only one complete observation. We did not impute missing values in the assembled panel; each model used complete cases for its stated variables. The final analytic key contained no duplicate country-year records. For a separate cross-sectional construct comparison, we downloaded the WHO Global Digital Health Monitor 2023 country maturity phase and seven domain scores (35); these data were not entered into the longitudinal outcome models.

### 2.3 Outcomes

The principal outcome was the WHO UHC service coverage index (SDG 3.8.1), scaled from 0 to 100 and constructed from tracer indicators spanning reproductive, maternal, newborn and child health; infectious diseases; noncommunicable diseases; and service capacity and access (2, 3, 32). Annual country values should not be interpreted as direct annual measurements. WHO source methods use modeled tracer series, linear interpolation between available years, constant extrapolation at series boundaries, and other imputations where necessary before constructing the composite (36). In the downloaded series, all values were integer-valued and 40.6% of adjacent country-year changes were zero. We therefore examined alternative estimands and a three-year UHC-change outcome. To examine sensitivity to modeled and interpolated annual values, we repeated the fixed-effects level model using the 2023 Global Monitoring Report reference years (2000, 2005, 2010, 2015, 2017, 2019, and 2021; 2000 dropped after lagging) and estimated annualized UHC change between adjacent reference years from start-year exposures.

Financial protection was represented by the percentages of the population spending more than 10% and more than 25% of household consumption or income on out-of-pocket health care. These data were sparse and survey-dependent: the 10% outcome contributed 775 observations from 149 countries and the 25% outcome contributed 774 observations from 148 countries. Secondary outcomes included DTP3, MCV1 and MCV2 immunization coverage; IHR/SPAR total capacity, surveillance and risk-communication scores; and log under-5 and neonatal mortality. These outcomes capture distinct domains and were not combined into a single effect scale.

### 2.4 Health-financing exposures

The principal financing exposures were domestic general government health expenditure as a percentage of current health expenditure (GGHE share) and household out-of-pocket expenditure as a percentage of current health expenditure (OOP share). These are compositional shares. When entered together, a coefficient represents a change in one share conditional on the other and on changes in the omitted remainder of current health expenditure; it is not an isolated increase in resources. We report overall and within-country correlations and estimate alternatives using government health expenditure as a percentage of GDP, log government health expenditure per capita, log current health expenditure per capita, public prepaid financing, and a private-burden measure.

### 2.5 Digital connectivity

The digital-connectivity composite contained three general infrastructure components: individuals using the Internet (% of population), mobile cellular subscriptions (per 100 people), and fixed broadband subscriptions (per 100 people). For each component, we calculated a pooled z score over the full 2000–2024 source panel before applying outcome-specific complete-case restrictions. The composite was the equal-weight arithmetic mean of available component z scores and required at least two of three components. We did not use *ad hoc* weights or outcome information in construction. Sensitivity analyses required all three components and estimated each component in its original units. Secure Internet servers and direct digital-health implementation measures were not included. Direct measures of adoption, interoperability, workforce capacity, and service integration were unavailable with comparable annual global coverage. We compared the 2023 connectivity composite with GDHM overall maturity and domain scores using Spearman correlations with 5,000 country-bootstrap confidence intervals. This cross-sectional analysis assessed the degree of overlap between general connectivity and dimensions of digital-health maturity.

### 2.6 Information environment and governance

Principal models included four one-year-lagged DSP components separately: government dissemination of false information, political-party dissemination of false information, online media pluralism, and online political fragmentation. For sensitivity and interaction analyses, we also created an adverse information-environment index. Each component was oriented so that higher values indicated a more adverse environment: government and party disinformation and fragmentation retained their direction, while pluralism was reversed. Components were pooled-standardized over the full 2000–2024 DSP source panel, equally weighted, and averaged only when all four were observed. This index captures a macro-level political-information environment; it is not a measure of health-misinformation prevalence or individual exposure.

Government effectiveness was measured using the official World Bank WGI estimate, a model-based indicator centered near zero and typically ranging from approximately −2.5 to 2.5, with higher values indicating stronger performance (30, 37). Additional controls were log GDP per capita at purchasing-power parity, urban population share, population aged 65 years or older, and access to at least basic drinking-water and sanitation services.

### 2.7 Statistical analysis

Principal linear models included country fixed effects and calendar-year fixed effects, with standard errors clustered by country. Country effects remove time-invariant national characteristics, including static health-system labels, while year effects remove shocks common across countries. They do not remove time-varying confounding, measurement error, reverse causality, or policy endogeneity. All continuous predictors were standardized within each analytic sample after lagging. We report overall, between-country, and within-country standard deviations to show the scale of identifying variation. Models based on different samples therefore have sample-specific SD units and should not be compared mechanically.

A one-year lag was selected as a short, consistent temporal ordering that preserved the breadth of the country panel; it was not assumed to represent the full latency of health-system change. Sensitivity analyses used two- and three-year exposure means, a three-year UHC-change outcome with exposures lagged 3 years, and health-workforce and hospital-bed controls. Digital-connectivity robustness analyses included within-between decomposition, region-by-year fixed effects, country-specific linear trends, first differences, a between-country model, and component-level models.

To examine temporal ordering, we estimated replacement and joint lag–lead models using connectivity measured one and 3 years after the outcome year. We also formed an equal-weight supply-capacity composite from pooled standardized physician, nurse/midwife, and hospital-bed densities, requiring at least two components, and compared matched-sample models before and after adjustment.

To assess the pandemic period, we estimated a digital-connectivity-by-2020-2023 interaction and separate models for 2001–2019 and 2020–2023. Income-group analyses compared high-income with non-high-income countries and then estimated the four World Bank income categories separately. Because the category-specific samples were smaller and standardization was sample-specific, these estimates were treated as descriptive heterogeneity rather than a formal causal test.

Interaction models examined GGHE share by adverse information environment, connectivity by government effectiveness, and connectivity by adverse information environment. All three UHC interactions used the same 3,522-observation complete-case sample and were also entered jointly. Secondary interaction models were re-estimated on common outcome-specific complete-case samples. Marginal effects of connectivity were calculated at moderator values of −1, 0, and +1 SD with 95% confidence intervals.

Benjamini–Hochberg adjustment was applied separately to 80 secondary main-effect tests, 20 common-sample interaction tests, and 10 adverse-information-index sensitivity tests (38). An exploratory FDR threshold of *q* ≤ 0.10 was used. Analyses were performed in Python using pandas, scipy, and statsmodels. For the eight GDHM construct correlations, 5,000-bootstrap confidence intervals and BH *q* values were reported.

## 3 Results

### 3.1 Sample and measurement

The principal UHC model included 3,522 country-years from 166 countries between 2001 and 2023 (Table 1). Mean UHC service coverage was 63.11 (overall SD 18.58; within-country SD 5.15). The connectivity composite had an overall SD of 0.868 source-panel units, a between-country SD of 0.676, and a within-country SD of 0.552; its median absolute annual change was 0.072. In contrast, the GGHE and OOP shares had overall SDs of 21.89 and 19.11 percentage points but within-country SDs of 6.328 and 6.246 percentage points, respectively. The official government-effectiveness estimate ranged from −2.21 to 2.35 in the principal sample. Figure 2 presents annual country means for UHC service coverage, health-financing shares, digital connectivity, and the adverse information environment.

**Table 1:** Descriptive statistics for the principal complete-case UHC sample.

Variable	*N*	Countries	Mean	Overall SD	Within-country SD	Median	Range
UHC service coverage index	3,522	166	63.112	18.576	5.153	69.000	14.00 to 92.00
Government health expenditure share (% current health expenditure)	3,522	166	49.406	21.887	6.328	50.446	0.79 to 90.22
Out-of-pocket expenditure share (% current health expenditure)	3,522	166	35.006	19.108	6.246	33.004	2.17 to 86.07
Digital connectivity composite (source-panel units)	3,522	166	−0.092	0.868	0.552	−0.225	−1.41 to 1.98
Government effectiveness estimate	3,522	166	−0.044	0.991	0.224	−0.200	−2.21 to 2.35
Log GDP per capita, PPP	3,522	166	9.389	1.176	0.187	9.477	6.57 to 11.89
Urban population (%)	3,522	166	57.341	22.248	3.375	59.077	8.04 to 100.00
Population aged 65 years or older (%)	3,522	166	8.030	5.913	1.141	5.404	0.86 to 29.40
At least basic drinking-water services (%)	3,522	166	84.810	18.095	4.244	92.690	18.76 to 100.00
At least basic sanitation services (%)	3,522	166	71.691	30.292	5.852	86.009	2.97 to 100.00

All predictors are lagged one year. The connectivity composite is shown in its source-panel standardized units; regression coefficients are reported per analytic-sample SD. Government effectiveness is the World Bank WGI estimate, typically ranging from approximately −2.5 to 2.5.

**Figure 2:**
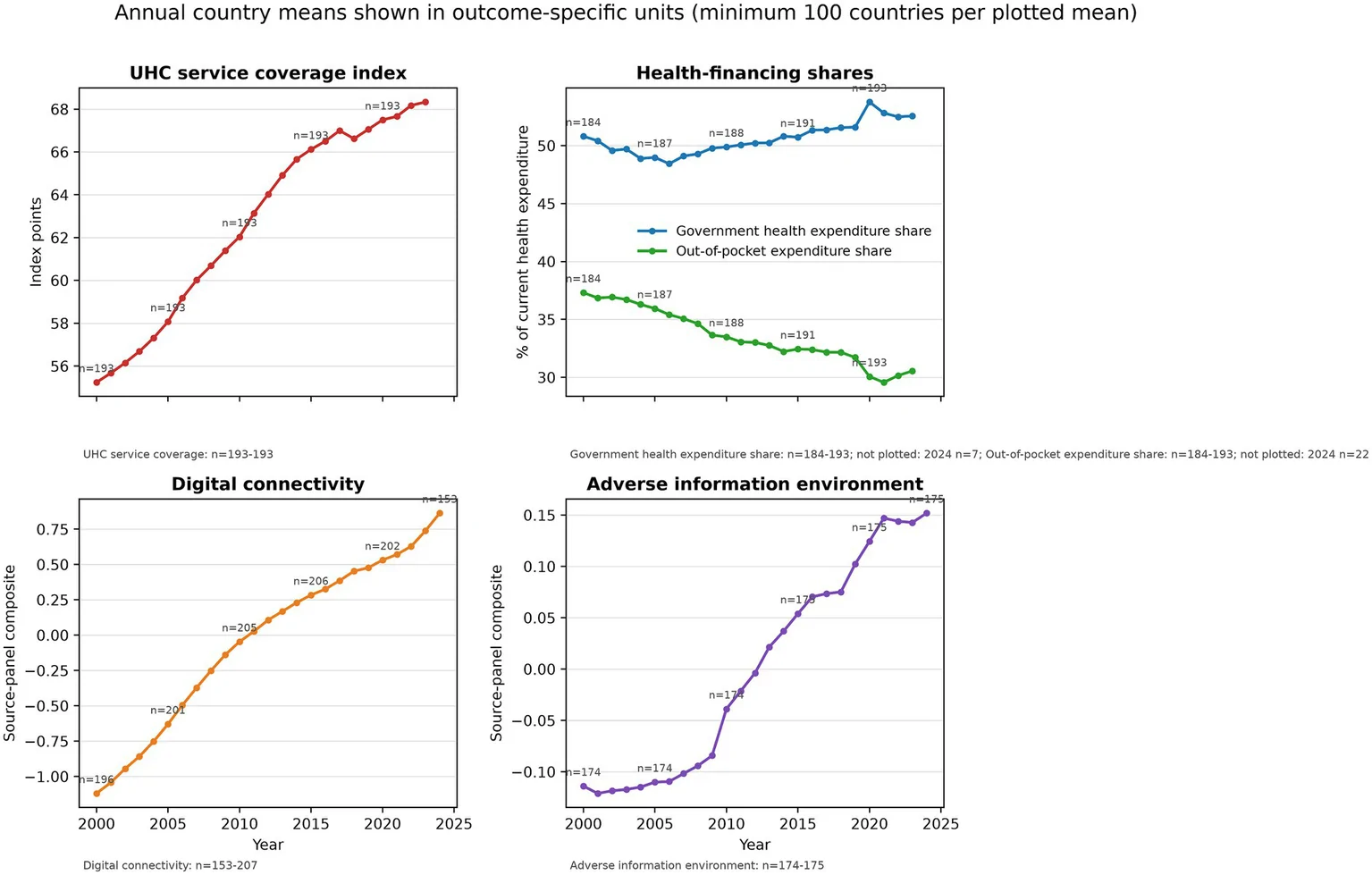
Annual country means in outcome-specific units. UHC service coverage, financing shares, digital connectivity, and the adverse information environment are shown in separate panels. Labels report selected annual country counts; panel notes report the count range and identify sparse financing years omitted from the plotted means. A minimum of 100 countries was required for a plotted annual mean.

### 3.2 Principal UHC associations

In the principal two-way fixed-effects model, a 1-SD higher one-year-lagged OOP share was associated with a 1.38-point lower UHC service coverage index (95% CI −2.39 to −0.36; *p* = 0.008). The GGHE share coefficient was −0.71 (95% CI −1.62 to 0.20; *p* = 0.128). A 1-SD higher general digital-connectivity composite was associated with a 2.98-point lower index (95% CI −4.05 to −1.91; *p* < 0.001). None of the four separate DSP information-environment indicators was independently associated with the UHC index. Basic sanitation was positively associated with the index; other controls had confidence intervals spanning zero (Table 2 and Figure 3).

**Table 2:** Principal two-way fixed-effects model of UHC service coverage.

One-year-lagged predictor	Coefficient (95% CI)	*p* value
Government health expenditure share	−0.71 (−1.62, 0.20)	0.128
Out-of-pocket expenditure share	−1.38 (−2.39, −0.36)	0.008
Digital connectivity	−2.98 (−4.05, −1.91)	<0.001
Government online disinformation	−0.44 (−1.16, 0.27)	0.221
Party online disinformation	0.47 (−0.31, 1.25)	0.241
Online media pluralism	0.30 (−0.50, 1.09)	0.464
Online political fragmentation	−0.24 (−1.13, 0.64)	0.592
Government effectiveness	0.88 (−0.40, 2.17)	0.177
Log GDP per capita, PPP	1.89 (−0.77, 4.56)	0.163
Urban population	1.75 (−0.88, 4.37)	0.192
Population aged 65+	−1.35 (−3.50, 0.80)	0.219
Basic drinking water	0.53 (−1.79, 2.85)	0.656
Basic sanitation	8.76 (6.17, 11.35)	<0.001

*N* = 3,522 country-years from 166 countries, 2001–2023. Models include country and year fixed effects and country-clustered standard errors. Continuous predictors were standardized within the analytic sample; coefficients therefore represent UHC index-point differences per 1 overall SD increase in each predictor.

**Figure 3:**
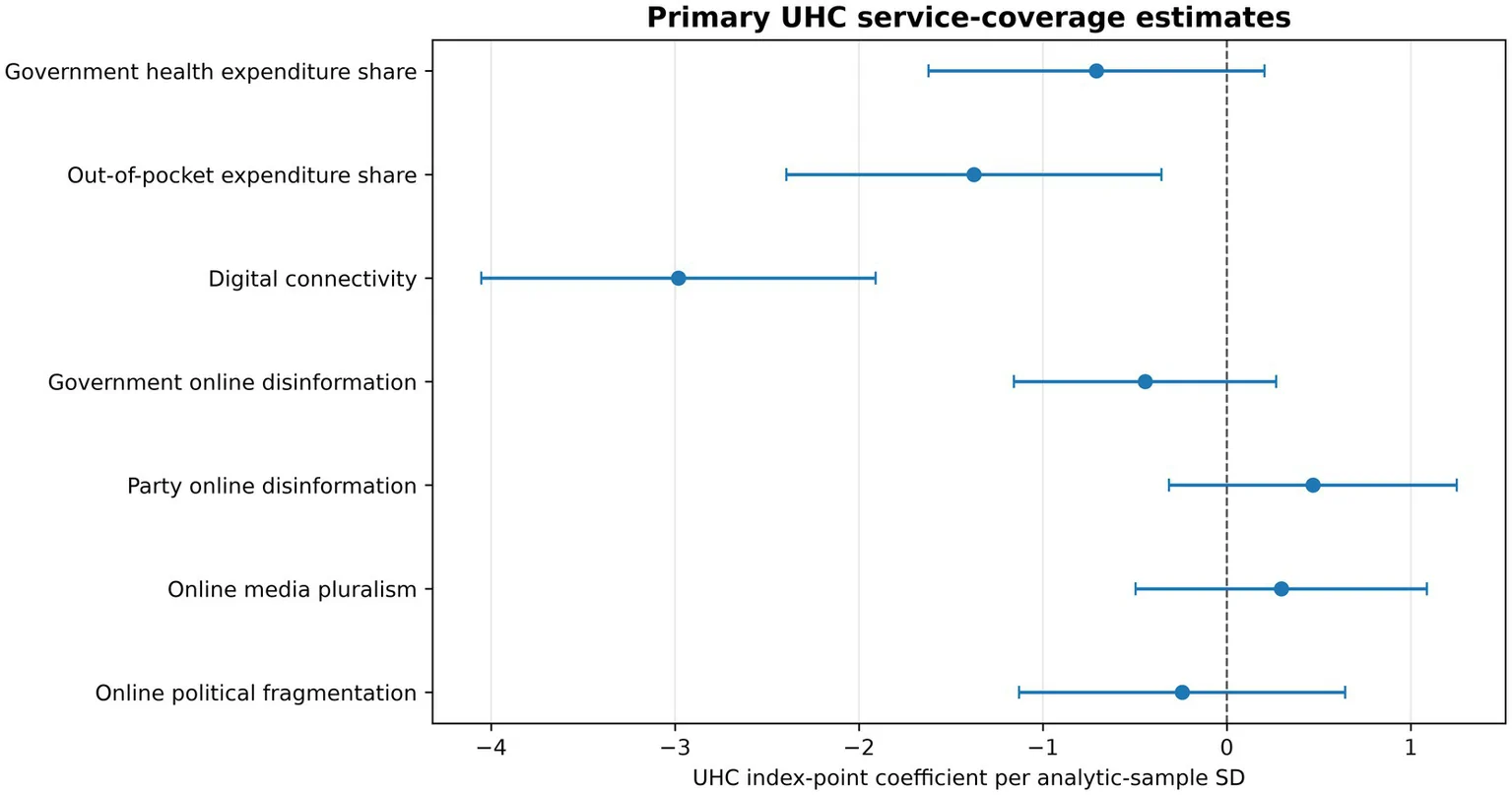
Principal two-way fixed-effects estimates for the UHC service coverage index. Points are coefficients per 1 analytic-sample SD increase in the one-year-lagged predictor; lines are 95% confidence intervals. The vertical line marks zero. The connectivity exposure measures general infrastructure, not digital-health adoption.

### 3.3 Scale, temporal, and estimand sensitivity

The negative connectivity direction was present in several specifications, but its magnitude depended on the estimand (Table 3 and Figure 4). The within-country deviation in the within-between model was −0.93 points (95% CI −1.94 to 0.09; *p* = 0.074), the country-trend estimate was −0.77 (95% CI −1.32 to −0.22), and the region-year estimate was −1.03 (95% CI −2.10 to 0.05; *p* = 0.061). The between-country coefficient was positive but imprecise. First differences were negative (−0.053 UHC change points per 1 SD of annual connectivity change; 95% CI −0.097 to −0.009). Because these models standardize different quantities and answer different questions, coefficient magnitudes should be interpreted within each estimand and scale.

**Table 3:** Digital-connectivity scale, temporal, COVID-19, and estimand sensitivity analyses.

Specification	Coefficient (95% CI)	*p* value	*N*	Countries
Primary two-way fixed effects	−2.98 (−4.05, −1.91)	<0.001	3,522	166
Region-year fixed effects	−1.03 (−2.10, 0.05)	0.061	3,522	166
Country-specific linear trends	−0.77 (−1.32, −0.22)	0.006	3,522	166
First differences	−0.05 (−0.10, −0.01)	0.019	3,198	166
Between-country mean model	1.22 (−2.20, 4.63)	0.486	166	166
Country mean in within-between model	0.89 (−2.38, 4.15)	0.593	3,522	166
Within-country deviation in within-between model	−0.93 (−1.94, 0.09)	0.074	3,522	166
Two-year exposure mean	−2.82 (−3.97, −1.67)	<0.001	3,198	166
Three-year exposure mean	−2.59 (−3.76, −1.42)	<0.001	3,020	166
Three-year UHC change on three-year-lagged exposures	−0.17 (−0.66, 0.32)	0.494	3,200	166
Digital connectivity x 2020–2023	−0.23 (−0.78, 0.31)	0.398	3,522	166
Digital connectivity slope in 2020–2023	−3.19 (−4.55, −1.83)	<0.001	3,522	166
Pre-COVID 2001–2019	−2.82 (−3.80, −1.85)	<0.001	2,875	166
COVID/post-COVID 2020–2023	0.13 (−1.63, 1.90)	0.883	647	163
Connectivity index requiring all three components	−2.14 (−3.26, −1.01)	<0.001	3,056	166
Internet users, per 10 percentage points	−0.89 (−1.14, −0.63)	<0.001	3,479	166
Mobile subscriptions, per 10 per 100 people	−0.21 (−0.32, −0.09)	<0.001	3,531	166
Fixed broadband, per 10 per 100 people	−0.39 (−1.05, 0.27)	0.250	3,110	166
WHO report-reference-year levels	−2.87 (−4.27, −1.48)	<0.001	966	166
WHO reference-interval annualized UHC change	−0.17 (−0.38, 0.04)	0.105	958	166
Joint lag–lead model: future connectivity t + 1	−0.87 (−1.91, 0.18)	0.103	3,335	166
Joint lag–lead model: future connectivity t + 3	−0.63 (−1.73, 0.47)	0.259	3,010	166
Two-of-three supply composite-adjusted model	−1.89 (−2.90, −0.89)	<0.001	2,490	165

Coefficients are not directly comparable across every row because the estimand and, for component models, the measurement unit differ. Internet use is per 10 percentage points; mobile and fixed broadband are per 10 subscriptions per 100 people; first differences use the standardized annual change in connectivity. The COVID-period interaction tests a slope difference rather than whether the period-specific slope itself differs from zero. The WHO reference-year level row remains in UHC index points; the reference-interval row is annualized UHC change. Future-connectivity rows are conditional future terms from joint lag–lead models, and the supply row uses its matched complete-case sample.

**Figure 4:**
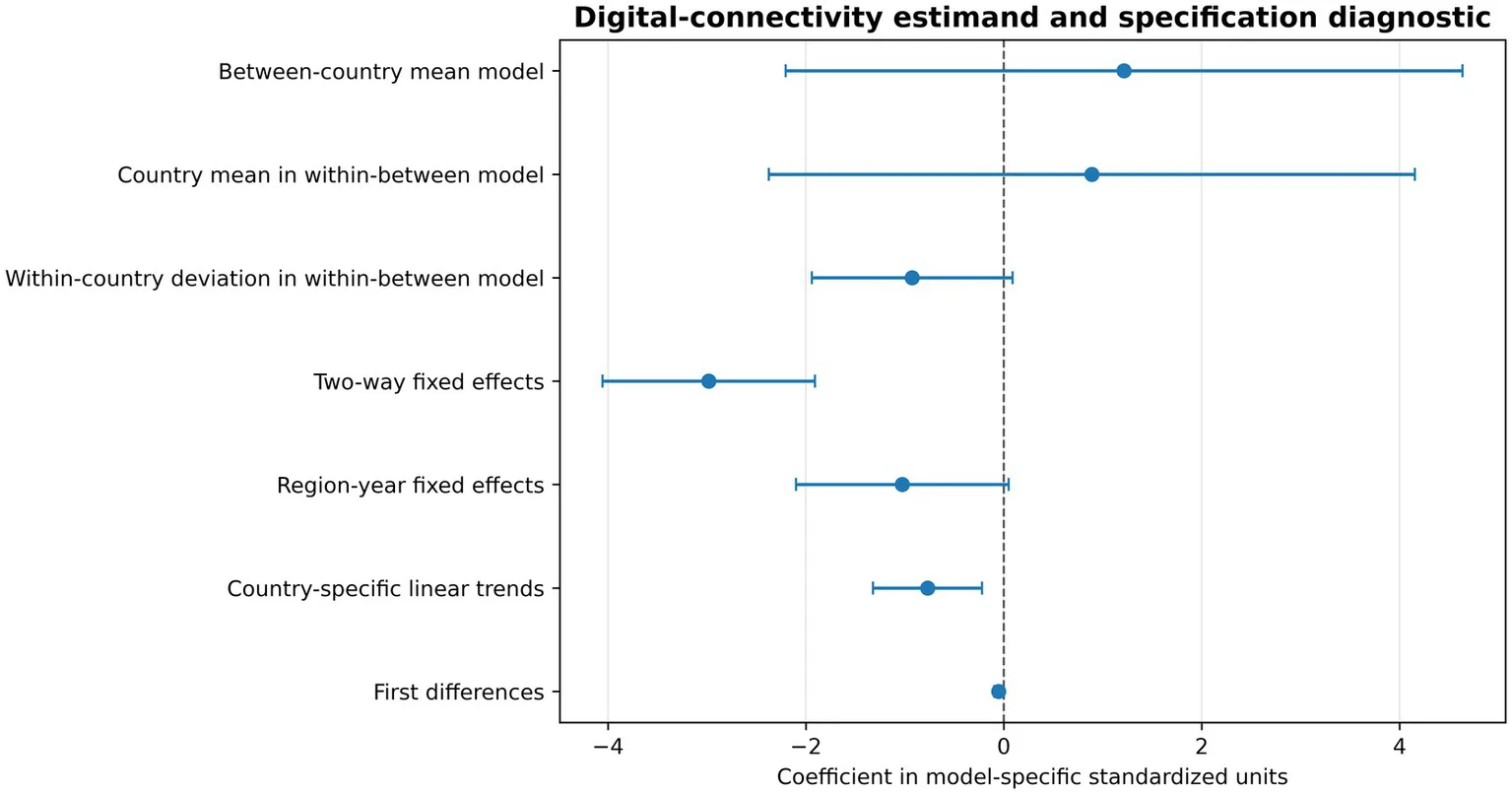
Digital-connectivity estimand diagnostic. The figure distinguishes between-country, within-between, two-way fixed-effects, region-year, country-trend, and first-difference estimates. Coefficients are model-specific standardized units and should be compared in direction and uncertainty, not treated as a single common causal scale.

Requiring all three connectivity components produced a coefficient of −2.14 (95% CI −3.26 to −1.01). In original units, 10 additional percentage points of Internet use were associated with −0.89 UHC points, and 10 additional mobile subscriptions per 100 people with −0.21 points; the fixed-broadband coefficient was imprecise. Two- and three-year exposure means remained negative, but three-year UHC change on three-year-lagged exposures was null (−0.17; 95% CI −0.66 to 0.32). The null change estimate is compatible with temporal mismatch, modeled outcome smoothness, residual confounding, or limited short-run service-coverage conversion.

Restricting the level model to WHO report-reference years yielded a similar connectivity coefficient (−2.87; 95% CI −4.27 to −1.48; *N* = 966). In contrast, start-year connectivity was not associated with annualized UHC change between adjacent reference years (−0.17 points per year; 95% CI −0.38 to 0.04; *p* = 0.105; *N* = 958). In joint lag–lead models, the future t + 1 (−0.87; 95% CI −1.91 to 0.18) and t + 3 terms (−0.63; 95% CI −1.73 to 0.47) were null after lagged connectivity was included. Correlations between lagged and future connectivity were 0.987 and 0.964, respectively, indicating limited temporal separation between the terms (Table 3).

The 2023 GDHM construct comparison included 151 countries for overall maturity; domain-specific sample sizes ranged from 62 to 151 countries. Connectivity correlated strongly with overall digital-health maturity (rho = 0.73), leadership and governance (rho = 0.69), legislation, policy, and compliance (rho = 0.69), and infrastructure (rho = 0.73), but only weakly to moderately with strategy and investment (rho = 0.16), workforce (rho = 0.32), standards and interoperability (rho = 0.20), and services and applications (rho = 0.28). The domain-specific correlations indicate uneven overlap between general connectivity and dimensions of digital-health maturity.

### 3.4 COVID-19 period and development context

The connectivity-by-2020-2023 interaction was −0.23 (95% CI −0.78 to 0.31; *p* = 0.398), providing no statistical evidence that the slope changed after 2020. The estimated post-2020 slope within the interaction model remained negative. Separate-period models yielded −2.82 (95% CI −3.80 to −1.85) in 2001–2019 and 0.13 (95% CI −1.63 to 1.90) in 2020–2023, but the latter used only 4 years and had substantially less within-country information. The nonsignificant interaction, rather than the apparent difference between separately estimated coefficients, is the relevant test of structural change.

The high-income estimate was 0.06 (95% CI −0.94 to 1.07), whereas the non-high-income estimate was −3.25 (95% CI −4.37 to −2.12). Estimates within the low-, lower-middle-, and upper-middle-income categories were smaller and individually imprecise. This pattern is compatible with heterogeneous conversion capacity, but it does not establish that income or institutional capacity causally modifies the association because the group models use different samples, variation, and standardization.

### 3.5 Financing composition and health-system supply

The overall correlation between GGHE and OOP shares was −0.75, and the within-country demeaned correlation was −0.63, underscoring their compositional dependence. Alternative financing models showed a positive association for log current health expenditure per capita (2.28; 95% CI 0.33 to 4.24) but not for government expenditure as a percentage of GDP, log government expenditure per capita, or public prepaid share. The connectivity coefficient remained negative across these alternatives. These estimates describe reallocations within financing structures and should not be interpreted as isolated policy effects.

Direct health-system supply indicators had substantially more missingness. On the common 1,826-observation sample, the base connectivity estimate was −0.32 (95% CI −1.21 to 0.58), and adding physician, nursing, and hospital-bed density changed it little (−0.41; 95% CI −1.26 to 0.43). Thus, the attenuation relative to the full sample primarily reflected sample composition, not statistical adjustment for these supply measures. The available indicators also do not capture pooling architecture, purchaser-provider relationships, primary-care organization, procurement, interoperability, or implementation quality. An expanded two-of-three supply composite retained 2,490 observations from 165 countries. On this matched sample, the connectivity coefficient was −1.77 (95% CI −2.81 to −0.74) before and −1.89 (95% CI −2.90 to −0.89) after supply adjustment; the supply composite was positively associated with UHC (1.34; 95% CI 0.19 to 2.49).

### 3.6 Exploratory secondary and interaction results

After FDR control across 80 secondary main-effect tests, four associations had q < =0.10 (Table 4). Connectivity was associated with lower DTP3 coverage (−4.62 percentage points; 95% CI −7.17 to −2.07; *q* = 0.010), lower MCV1 coverage (−3.74; 95% CI −6.17 to −1.30; *q* = 0.053), and lower neonatal mortality on the log scale (−0.127; 95% CI −0.190 to −0.065; *q* = 0.004), equivalent to approximately 12.0% lower mortality. GGHE share was associated with higher MCV2 coverage (12.86; 95% CI 6.34 to 19.38; *q* = 0.004). The nominal association between GGHE share and catastrophic expenditure at the 10% threshold was −2.21 percentage points (95% CI −4.35 to −0.06; *p* = 0.044) but did not survive FDR control (*q* = 0.269).

**Table 4:** Selected exploratory secondary and common-sample interaction results.

Outcome	Analysis	Coefficient (95% CI)	*p*	BH *q*	*N*	Countries
Catastrophic OOP, 10% threshold	Main effect: Government health expenditure share	−2.207 (−4.353, −0.062)	0.044	0.269	775	149
DTP3 coverage	Main effect: Digital connectivity	−4.618 (−7.169, −2.067)	<0.001	0.010	3,668	166
MCV1 coverage	Main effect: Digital connectivity	−3.737 (−6.172, −1.302)	0.003	0.053	3,668	166
MCV2 coverage	Main effect: Government health expenditure share	12.859 (6.336, 19.382)	<0.001	0.004	3,668	166
Neonatal mortality	Main effect: Digital connectivity	−0.127 (−0.190, −0.065)	<0.001	0.004	3,668	166
MCV1 coverage	Interaction: Connectivity x Government effectiveness	1.500 (0.500, 2.500)	0.003	0.022	3,668	166
MCV2 coverage	Interaction: Connectivity x Government effectiveness	6.838 (3.229, 10.448)	<0.001	0.002	3,668	166
MCV2 coverage	Interaction: Connectivity x Adverse information environment	−5.977 (−9.125, −2.830)	<0.001	0.002	3,668	166

All secondary and conditional analyses were treated as exploratory. Benjamini-Hochberg *q* values were calculated across 80 main-effect tests and separately across 20 common-sample interaction tests. The catastrophic-expenditure estimate was nominally significant but did not survive FDR control.

All three UHC interaction terms were null on the same 3,522-observation complete-case sample. On common secondary samples, three immunization interactions survived FDR control. Higher government effectiveness attenuated the negative connectivity slope for MCV1 and MCV2; at +1 SD of government effectiveness, the MCV2 marginal slope was 0.66 (95% CI −7.83 to 9.15), not a statistically positive effect. A more adverse information environment steepened the MCV2 slope: the marginal effect changed from 3.18 (95% CI −5.33 to 11.69) at −1 SD to −8.78 (95% CI −16.24 to −1.31) at +1 SD. These results support conditionality for selected immunization outcomes, not a general claim that governance reverses the UHC association (Figure 5).

**Figure 5:**
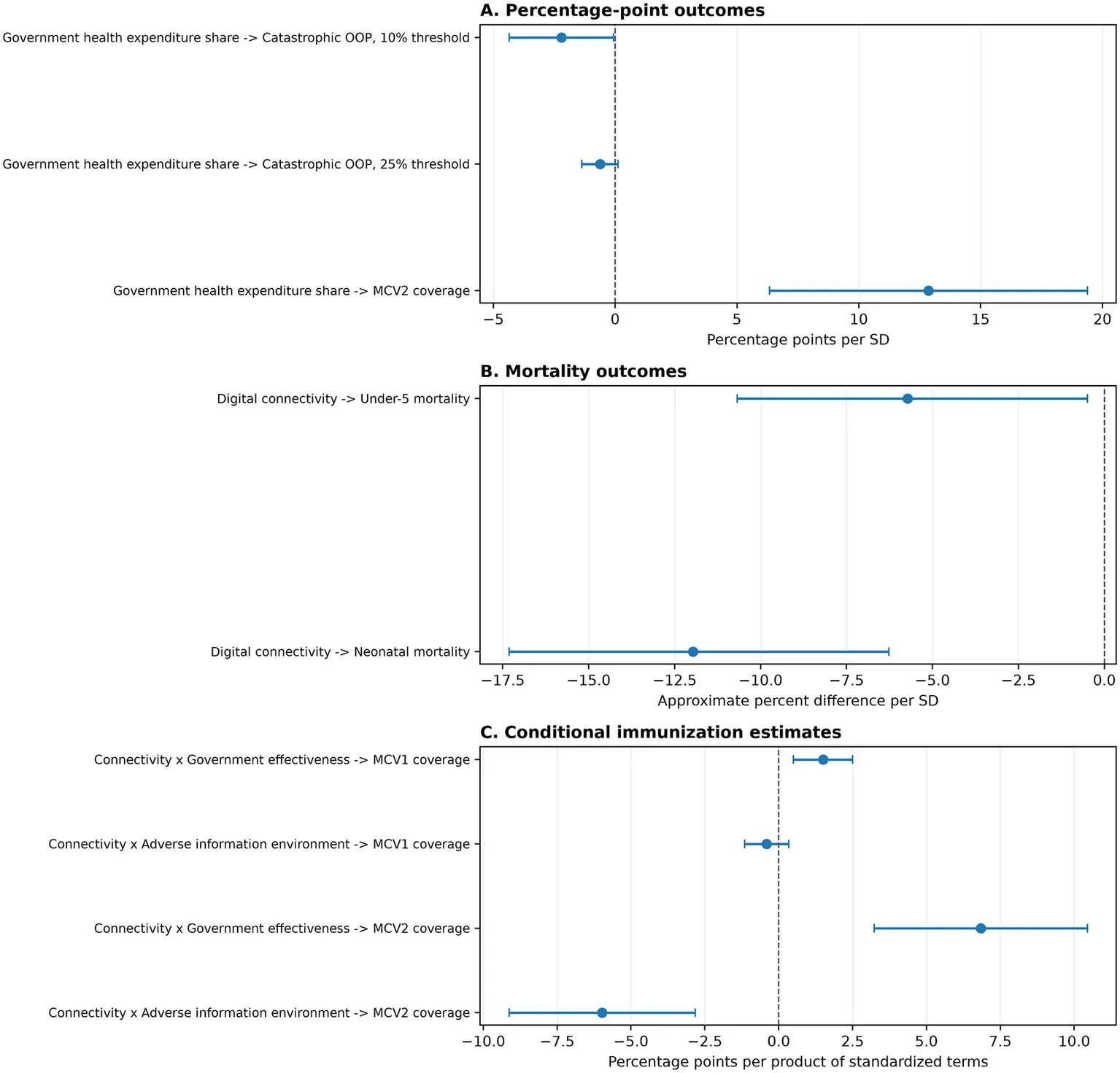
Selected exploratory secondary associations and common-sample interactions in outcome-specific units. **(A)** Associations of government health expenditure share with catastrophic out-of-pocket expenditure at the 10% and 25% thresholds and MCV2 coverage, expressed in percentage points per 1-SD increase in the exposure. **(B)** Associations of digital connectivity with under-5 and neonatal mortality, expressed as percentage differences per 1-SD increase in connectivity, calculated as 100 × [exp(β) − 1]. **(C)** Interactions of digital connectivity with government effectiveness and the adverse information environment for MCV1 and MCV2 coverage, expressed in percentage points per unit increase in the product of the standardized exposure and moderator. Points indicate estimates and horizontal lines indicate 95% confidence intervals. All secondary analyses are exploratory.

## 4 Discussion

### 4.1 Principal interpretation

The results support a conditional-conversion interpretation. Within the countries and years represented in the principal model, increases in general connectivity were not accompanied by short-term gains in the UHC service coverage index. The association was negative in the principal two-way fixed-effects model, smaller in several alternative estimands, and null for three-year UHC change. Because the exposure captures general infrastructure rather than implemented digital-health services, the findings concern the conversion of connectivity into service coverage.

The conditional conversion framework helps organize this distinction. Connectivity can support electronic records, telemedicine, decision support, logistics, surveillance, and public communication only when those applications are financed, governed, procured, maintained, interoperable, usable by health workers, and integrated into service pathways (10, 11). The 2023 GDHM construct comparison sharpened this distinction: connectivity overlapped strongly with overall maturity, leadership and governance, legislation and policy, and infrastructure, but only weakly to moderately with strategy and investment, workforce, standards and interoperability, and services and applications. These correlations position general connectivity as an enabling-infrastructure measure rather than a proxy for all dimensions of digital-health maturity.

### 4.2 Health-system organization and development

Health-system organization remains an important unmeasured source of heterogeneity. Similar expenditure shares can arise from different revenue sources, pooling arrangements, purchaser-provider relationships, and degrees of segmentation. Classic typologies are often restricted to OECD countries and may incorrectly assign hybrid systems to a single category (22, 23). Static categories would also be absorbed by country fixed effects. On matched samples, connectivity estimates were similar before and after adjustment for individual supply measures or the two-of-three supply composite; these indicators represent input capacity rather than organizational architecture. The high-income/non-high-income contrast is compatible with stronger absorptive capacity in wealthier settings, but the smaller and imprecise four-category estimates counsel against a deterministic development-gradient claim.

Conversion capacity in this study is therefore a theoretical construct only partly operationalized by government effectiveness, financing structure, income, water and sanitation access, health-worker and bed density, and information-environment measures. Important unmeasured elements include primary-care organization, interoperability, electronic-record maturity, digital governance, workforce competencies, procurement and maintenance capacity, regulatory and data-governance maturity, platform usability, clinical workflow integration, and trust. Future research should link connectivity to longitudinal digital-health maturity and implementation indicators as their coverage improves.

### 4.3 COVID-19 and temporal sensitivity

COVID-19 accelerated telemedicine and other forms of digitally mediated care (15, 24, 25), but we found no statistically detectable post-2020 change in the UHC connectivity slope. The short post-2020 window, the slow-moving and partly modeled UHC index, and the concentration of pandemic-era digital changes in specific services limit the sensitivity of this test. Process outcomes such as appointment access, referral completion, continuity, surveillance timeliness, or telemedicine use may respond more rapidly than the aggregate index. Annualized UHC change between WHO report-reference years was also not associated with connectivity, consistent with the three-year-change model.

### 4.4 Financing and financial protection

The inverse OOP-UHC association is consistent with the central role of prepayment and pooling in UHC (5). However, financing shares are compositional, and fixed-effects coefficients capture within-country reallocations conditional on other included components. The nominal GGHE association with catastrophic expenditure at the 10% threshold is substantively plausible but did not survive multiple-testing control and came from a sparse, irregular survey panel. It should be considered suggestive rather than confirmatory. The financial-protection outcomes remain conceptually essential because service coverage alone does not show whether care is affordable.

### 4.5 Domain-specific outcomes and information environments

The secondary results were heterogeneous rather than uniformly favorable or unfavorable. Connectivity was associated with lower neonatal mortality but also lower DTP3 and MCV1 coverage after FDR control. Such divergence may reflect different outcome time scales, reporting systems, confounding, or sample structure. It may also indicate that general connectivity supports some information, coordination, or socioeconomic pathways without ensuring routine immunization delivery. The mortality and immunization associations should therefore be interpreted as separate exploratory findings.

The immunization interactions offer limited evidence that institutional and information contexts matter. Higher government effectiveness attenuated negative MCV1 and MCV2 slopes, while a more adverse information environment steepened the MCV2 slope. Neither result generalized to the composite UHC outcome. DSP variables describe broad political communication, pluralism, and fragmentation rather than vaccine misinformation intensity, health-news attention, or individual beliefs (17–21, 27). Mechanistic claims require health-specific media and platform data.

### 4.6 Equity and the digital divide

National connectivity averages can rise while rural, low-income, older, disabled, linguistically marginalized, or otherwise disadvantaged groups remain excluded. Digital services can also require devices, stable broadband, literacy, accessible design, identity systems, and trust. WHO evidence warns that unequal access, use, and engagement can widen existing health inequities (39). Because UHC is population-wide, infrastructure expansion concentrated among already advantaged groups may have little effect on national equity even when the country mean rises. The present panel cannot test within-country digital divides or distributional UHC effects.

### 4.7 Strengths and limitations

Strengths include the large longitudinal multi-source panel, explicit separation of service coverage and financial protection, transparent characterization of UHC index construction, official governance scale, country and year fixed effects, clustered inference, alternative lag and estimand checks, COVID-period testing, common-sample interaction models, marginal effects, and complete FDR-controlled reporting of exploratory outcomes. Sensitivity analyses also included WHO report-reference-year and interval-change models, lag–lead diagnostics, a GDHM construct comparison, and expanded supply-capacity adjustment.

Several limitations should be considered. First, the ecological observational design cannot establish causality. Lagging and fixed effects do not eliminate reverse causality, time-varying economic or institutional confounding, policy endogeneity, or differential measurement error. Second, connectivity does not measure digital-health adoption, quality, interoperability, utilization, or implementation fidelity. Third, health-system architecture and most elements of conversion capacity are unmeasured. Fourth, national averages conceal within-country inequality. Fifth, the UHC composite contains modeled, interpolated, extrapolated, and integer-valued annual estimates and may be insensitive to early or domain-specific change. Sixth, financial-protection, preparedness, and health-system supply outcomes have sparse or selective coverage. Seventh, sample-specific standardization limits coefficient comparison across models. Eighth, multiple secondary analyses remain exploratory despite FDR control. Finally, DSP variables are macro-level political-information measures and cannot identify exposure to health misinformation. Accordingly, the principal coefficient represents a model-dependent country-level association between general connectivity and UHC service coverage.

## 5 Conclusion

Across a 166-country panel, greater out-of-pocket financing was associated with lower UHC service coverage. General digital connectivity was negatively associated with UHC levels in the principal model, but the magnitude varied across estimands, and both three-year and WHO reference-interval change models were null. The GDHM comparison showed uneven overlap between connectivity and digital-health maturity domains, while domain-specific health associations were heterogeneous. Together, the findings suggest that connectivity alone is insufficient to improve UHC; its contribution likely depends on financing, governance, health-system organization, interoperable implementation, workforce readiness, trustworthy communication, and equitable access. Future research should link longitudinal measures of digital-health adoption and implementation to subnational service-coverage and equity outcomes.

## Data Availability

The datasets presented in this study can be found in online repositories. The names of the repository/repositories and accession number(s) can be found at: https://doi.org/10.5281/zenodo.21200057.
