# Supplementary material for: Health financing, digital connectivity, and universal health coverage: a country-year panel study
Source: Front Public Health. 2026 Oct 9;14:1928583. doi: 10.3389/fpubh.2026.1928583 (PMC13690777; doi:10.3389/fpubh.2026.1928583)
Supplement: Supplementary file 1 [file Table_1.docx]

Supplementary Material

**Health financing, digital connectivity, and universal health coverage: a country-year panel study**

Supplementary results for UHC model diagnostics, digital-health construct comparisons, and secondary outcomes.

**Table S1. UHC interactions and temporal-ordering diagnostics**

| **Model / term** | **Coefficient (95% CI)** | **p** | **N** |
| --- | --- | --- | --- |
| GGHE share × adverse information | 0.227 (-0.373, 0.827) | 0.459 | 3,522 |
| Connectivity × government effectiveness | 0.318 (-0.168, 0.804) | 0.200 | 3,522 |
| Connectivity × adverse information | -0.208 (-0.594, 0.179) | 0.293 | 3,522 |
| Future-only t+1: future connectivity | -2.69 (-3.80, -1.58) | <0.001 | 3,355 |
| Joint t+1: lagged connectivity (t−1) | -2.23 (-3.25, -1.22) | <0.001 | 3,335 |
| Joint t+1: future connectivity (t+1) | -0.87 (-1.91, 0.18) | 0.103 | 3,335 |
| Future-only t+3: future connectivity | -1.95 (-3.16, -0.74) | 0.002 | 3,031 |
| Joint t+3: lagged connectivity (t−1) | -2.53 (-3.46, -1.60) | <0.001 | 3,010 |
| Joint t+3: future connectivity (t+3) | -0.63 (-1.73, 0.47) | 0.259 | 3,010 |

Note. All models include country and year fixed effects, the principal covariates, and country-clustered standard errors; each includes 166 countries. The first three rows are separate interaction models (2001–2023). Continuous predictors are standardized within the analytic sample. GGHE = government health expenditure; UHC = universal health coverage. Joint lag–lead correlations are 0.987 (t+1) and 0.964 (t+3).

**Table S2. Connectivity correlations with 2023 WHO GDHM measures**

| **WHO GDHM measure** | **Spearman ρ (95% CI)** | **p** | **BH q** | **Countries** |
| --- | --- | --- | --- | --- |
| Overall digital-health maturity phase | 0.73 (0.64, 0.81) | <0.001 | <0.001 | 151 |
| Leadership and governance | 0.69 (0.58, 0.78) | <0.001 | <0.001 | 137 |
| Strategy and investment | 0.16 (-0.12, 0.42) | 0.208 | 0.208 | 62 |
| Legislation, policy, and compliance | 0.69 (0.58, 0.77) | <0.001 | <0.001 | 151 |
| Workforce | 0.32 (0.07, 0.55) | 0.010 | 0.016 | 63 |
| Standards and interoperability | 0.20 (-0.06, 0.44) | 0.112 | 0.128 | 63 |
| Infrastructure | 0.73 (0.62, 0.81) | <0.001 | <0.001 | 150 |
| Services and applications | 0.28 (0.03, 0.50) | 0.027 | 0.036 | 63 |

Note. GDHM = Global Digital Health Monitor. Confidence intervals use 5,000 country-level bootstrap samples. Benjamini–Hochberg (BH) q values cover all eight measures. Country counts vary with measure availability.

**Table S3. Financing and connectivity associations with secondary outcomes**

| **Outcome** | **Exposure** | **Coefficient (95% CI)** | **p** | **BH q** | **N / countries** |
| --- | --- | --- | --- | --- | --- |
| Catastrophic OOP, 10% threshold | GGHE share | -2.207 (-4.353, -0.062) | 0.044 | 0.269 | 775 / 149 |
|  | OOP share | -0.298 (-2.413, 1.816) | 0.782 | 0.958 |  |
|  | Connectivity | 0.853 (-0.599, 2.305) | 0.250 | 0.736 |  |
| Catastrophic OOP, 25% threshold | GGHE share | -0.622 (-1.366, 0.122) | 0.101 | 0.353 | 774 / 148 |
|  | OOP share | -0.319 (-1.063, 0.424) | 0.400 | 0.797 |  |
|  | Connectivity | -0.043 (-0.485, 0.399) | 0.848 | 0.971 |  |
| DTP3 coverage | GGHE share | 0.430 (-1.525, 2.385) | 0.666 | 0.945 | 3,668 / 166 |
|  | OOP share | -1.069 (-2.918, 0.781) | 0.258 | 0.736 |  |
|  | Connectivity | -4.618 (-7.169, -2.067) | <0.001 | 0.010 |  |
| MCV1 coverage | GGHE share | 0.494 (-1.817, 2.805) | 0.675 | 0.945 | 3,668 / 166 |
|  | OOP share | -0.289 (-2.231, 1.654) | 0.771 | 0.958 |  |
|  | Connectivity | -3.737 (-6.172, -1.302) | 0.003 | 0.053 |  |
| MCV2 coverage | GGHE share | 12.859 (6.336, 19.382) | <0.001 | 0.004 | 3,668 / 166 |
|  | OOP share | 5.927 (-0.620, 12.474) | 0.076 | 0.338 |  |
|  | Connectivity | -3.507 (-11.281, 4.267) | 0.377 | 0.797 |  |
| IHR/SPAR total capacity | GGHE share | 1.853 (-2.604, 6.311) | 0.415 | 0.797 | 619 / 162 |
|  | OOP share | -1.756 (-5.363, 1.852) | 0.340 | 0.797 |  |
|  | Connectivity | 2.230 (-2.340, 6.799) | 0.339 | 0.797 |  |
| Risk communication capacity | GGHE share | 3.761 (-6.062, 13.584) | 0.453 | 0.805 | 474 / 162 |
|  | OOP share | -3.868 (-13.443, 5.706) | 0.428 | 0.797 |  |
|  | Connectivity | 10.321 (-11.982, 32.625) | 0.364 | 0.797 |  |
| Surveillance capacity | GGHE share | 1.282 (-7.364, 9.928) | 0.771 | 0.958 | 474 / 162 |
|  | OOP share | -2.104 (-8.596, 4.389) | 0.525 | 0.841 |  |
|  | Connectivity | 5.337 (-10.055, 20.729) | 0.497 | 0.841 |  |
| Under-5 mortality | GGHE share | 0.011 (-0.041, 0.062) | 0.691 | 0.945 | 3,668 / 166 |
|  | OOP share | 0.016 (-0.033, 0.065) | 0.522 | 0.841 |  |
|  | Connectivity | -0.059 (-0.113, -0.005) | 0.032 | 0.255 |  |
| Neonatal mortality | GGHE share | -0.001 (-0.050, 0.048) | 0.964 | 0.987 | 3,668 / 166 |
|  | OOP share | 0.003 (-0.043, 0.049) | 0.905 | 0.987 |  |
|  | Connectivity | -0.127 (-0.190, -0.065) | <0.001 | 0.004 |  |

Note. Adjusted country and year fixed-effects models with country-clustered standard errors. Coefficients are per analytic-sample SD of the one-year-lagged exposure. GGHE and OOP are shares of current health expenditure. Outcomes use percentage points (catastrophic spending and vaccination), capacity-score points (IHR/SPAR), or log mortality rates. BH q values retain the original adjustment across all 80 secondary main-effect tests; they are not recalculated for the 30 estimates shown here.

Note. OOP = out-of-pocket expenditure; DTP3 = third dose of diphtheria–tetanus–pertussis vaccine; MCV1/MCV2 = first/second dose of measles-containing vaccine; IHR/SPAR = International Health Regulations State Party Self-Assessment Annual Reporting. Catastrophic-spending samples include 26 (10% threshold) and 25 (25% threshold) countries with one observation; each has 123 countries with at least two observations.

**Table S4. Connectivity interactions for secondary outcomes**

| **Outcome** | **Moderator** | **Interaction (95% CI)** | **p** | **BH q** | **N / countries** |
| --- | --- | --- | --- | --- | --- |
| Catastrophic OOP, 10% threshold | Government effectiveness | 0.455 (-0.475, 1.386) | 0.338 | 0.614 | 775 / 149 |
|  | Adverse information environment | -0.086 (-0.714, 0.542) | 0.788 | 0.851 |  |
| Catastrophic OOP, 25% threshold | Government effectiveness | 0.205 (-0.066, 0.476) | 0.138 | 0.553 | 774 / 148 |
|  | Adverse information environment | -0.032 (-0.236, 0.171) | 0.756 | 0.851 |  |
| DTP3 coverage | Government effectiveness | 0.662 (-0.406, 1.731) | 0.225 | 0.614 | 3,668 / 166 |
|  | Adverse information environment | -0.158 (-0.890, 0.574) | 0.673 | 0.851 |  |
| MCV1 coverage | Government effectiveness | 1.500 (0.500, 2.500) | 0.003 | 0.022 | 3,668 / 166 |
|  | Adverse information environment | -0.408 (-1.157, 0.341) | 0.285 | 0.614 |  |
| MCV2 coverage | Government effectiveness | 6.838 (3.229, 10.448) | <0.001 | 0.002 | 3,668 / 166 |
|  | Adverse information environment | -5.977 (-9.125, -2.830) | <0.001 | 0.002 |  |
| IHR/SPAR total capacity | Government effectiveness | 0.401 (-3.268, 4.069) | 0.830 | 0.851 | 619 / 162 |
|  | Adverse information environment | 0.763 (-1.901, 3.426) | 0.575 | 0.851 |  |
| Risk communication capacity | Government effectiveness | -2.462 (-17.443, 12.519) | 0.747 | 0.851 | 474 / 162 |
|  | Adverse information environment | -0.715 (-8.152, 6.722) | 0.851 | 0.851 |  |
| Surveillance capacity | Government effectiveness | -1.299 (-10.289, 7.691) | 0.777 | 0.851 | 474 / 162 |
|  | Adverse information environment | -2.280 (-6.774, 2.215) | 0.320 | 0.614 |  |
| Under-5 mortality | Government effectiveness | 0.006 (-0.021, 0.032) | 0.675 | 0.851 | 3,668 / 166 |
|  | Adverse information environment | -0.014 (-0.039, 0.012) | 0.296 | 0.614 |  |
| Neonatal mortality | Government effectiveness | 0.020 (-0.009, 0.048) | 0.176 | 0.586 | 3,668 / 166 |
|  | Adverse information environment | -0.027 (-0.056, 0.002) | 0.063 | 0.316 |  |

Note. Each row reports the interaction between digital connectivity and the listed moderator in an adjusted country and year fixed-effects model with country-clustered standard errors. Connectivity and the moderator are standardized before multiplication. Each outcome uses a common sample across the two moderators. BH q values cover all 20 interaction tests; the exploratory false discovery rate threshold is q ≤ 0.10. Outcome units, abbreviations, and sample-count conventions are as in Table S3.
